# Aphids evolved novel secreted proteins for symbiosis with bacterial endosymbiont

**DOI:** 10.1098/rspb.2012.1952

**Published:** 2013-01-07

**Authors:** Shuji Shigenobu, David L. Stern

**Affiliations:** 1 NIBB Core Research Facilities, National Institute for Basic Biology, Myodaiji, Okazaki 444-8585, Japan; 2 PRESTO, JST, 4-1-8 Honcho Kawaguchi, Saitama 332-0012, Japan; 3 Howard Hughes Medical Institute, Department of Ecology and Evolution, Princeton University, Princeton, NJ 08450, USA

**Keywords:** symbiosis, aphid, antimicrobial peptide, deep sequencing, *Buchnera*

## Abstract

Aphids evolved novel cells, called bacteriocytes, that differentiate specifically to harbour the obligatory mutualistic endosymbiotic bacteria *Buchnera aphidicola*. The genome of the host aphid *Acyrthosiphon pisum* contains many orphan genes that display no similarity with genes found in other sequenced organisms, prompting us to hypothesize that some of these orphan genes are related to lineage-specific traits, such as symbiosis. We conducted deep sequencing of bacteriocytes mRNA followed by whole mount *in situ* hybridizations of over-represented transcripts encoding aphid-specific orphan proteins. We identified a novel class of genes that encode small proteins with signal peptides, which are often cysteine-rich, that are over-represented in bacteriocytes. These genes are first expressed at a developmental time point coincident with the incorporation of symbionts strictly in the cells that contribute to the bacteriocyte and this bacteriocyte-specific expression is maintained throughout the aphid's life. The expression pattern suggests that recently evolved secretion proteins act within bacteriocytes, perhaps to mediate the symbiosis with beneficial bacterial partners, which is reminiscent of the evolution of novel cysteine-rich secreted proteins of leguminous plants that regulate nitrogen-fixing endosymbionts.

## 1. Introduction

Approximately 200 Ma, aphids evolved novel cells, called bacteriocytes, that house the gamma-proteobacterial endosymbiont, *Buchnera aphidicola*. Physiological studies and whole-genome sequence analysis of *Acyrthosiphon pisum* and *Buchnera* have revealed an intimate metabolic collaboration between partners. *Buchnera* provides the host with nutrition such as amino acids that aphids cannot synthesize and that are deficient in plant phloem sap, aphids' sole dietary component (reviewed in [1,2]). The aphid–*Buchnera* association is obligate and mutualistic with neither partner being able to reproduce in the absence of the other [1,2]. The symbionts are passed directly from mother to offspring by transovarial transfer [3,4].

*Acyrthosiphon pisum*, with 34 604 predicted genes in its 464-Mb draft genome assembly, has the highest gene count of any insect sequenced to date, although a precise gene count awaits better genome assembly and further analyses [5]. This high gene count reflects both extensive gene duplications and the presence of aphid-specific orphan genes. Orphan genes, which display no significant similarity with genes identified in other sequenced organisms, comprise 20 per cent of the total number of genes in the pea aphid genome [5]. The functions of these orphan genes are unknown, but their evolution may be related to the evolution of lineage-specific traits in aphids, such as symbiosis with *Buchnera*.

To understand the host's role in this symbiotic system, mRNA of the host bacteriocyte of the pea aphid *Acyrthosiphon pisum* has been assessed by expressed sequence tag (EST)-based transcriptome analyses [6,7] and, more recently, by deep-sequencing transcriptome analysis [8]. Many of the over-represented transcripts are associated with the utilization of essential amino acids, biosynthesis of non-essential amino acids and transport, which support the metabolic collaboration between the host and symbiont. In addition, laterally acquired genes of alpha-proteobacterial origin are also preferentially transcribed in bacteriocytes [6,9,10]. Orphan genes have not been the focus of previous studies.

In this study, we conducted deep sequencing of mRNAs isolated from bacteriocytes followed by whole mount *in situ* hybridization of selected over-represented genes and focused on aphid-specific orphan genes. Here, we report the identification of a novel class of genes that encode small proteins with signal peptides, which are often cysteine-rich, that are expressed exclusively in bacteriocytes. These genes are expressed first at a developmental time point coincident with the incorporation of symbionts and their bacteriocyte-specific expression is maintained throughout the aphid's life.

## 2. Material and methods

### (a) Aphids and RNA samples

*Acyrthosiphon pisum* strain LSR1 was used in this study. Parthenogenetic aphid clones were reared on broad bean, *Vicia faba,* in growth chambers with a long-day photoperiod (16 L : 8 D cycle) at 19°C. L3–L4 nymphs and young adults of parthenogenetic females were used to prepare bacteriocyte and whole body samples. More details on dissections and RNA extraction are provided in the electronic supplementary material, materials and methods.

### (b) Deep sequencing

RNA-seq libraries were constructed using Illumina mRNA-seq Sample Prep Kit (Illumina #RS-100-0801). The libraries were run for 35 or 36 cycles by The Genomics Core Facility at Princeton University and the Genomics Resource Center at Rockefeller University. RNA-seq reads have been deposited at the Short Read Archive (NCBI) under accession no. SRA026589. Details are described in the electronic supplementary material, materials and methods.

### (c) RNA-seq data analysis

Illumina reads from multiple runs were combined. We built a custom transcriptome reference set consisting of pea aphid official gene set v. 1.0 (OGS1) [5], which was downloaded from AphidBase (http://www.aphidbase.com/), and cDNA sequences overlooked from OGS1 [11], which amount to 34 639 gene models in total. The Illumina data were aligned to the custom transcriptome reference using Bowtie [12]. The Bowtie outputs were processed to obtain tag counts and reads per kilobase per million (RPKM) values using a custom script. Differential expression between libraries was assessed by the likelihood ratio test as implemented in the program DEGseq [13]. Secretion signal sequences were predicted by SignalP v. 3.0 [14]. Details of database search and gene set enrichment analysis are described in the electronic supplementary material, materials and methods.

### (d) Whole mount *in situ* hybridization

DIG-labelled RNA probes were generated from our *A. pisum* 50 k full-length cDNA collection [11]. Detailed procedures are described in the electronic supplementary material, materials and methods. Clone names used and associated EST accessions are shown in electronic supplementary material, table S1. Bacteriocytes and viviparous ovaries were dissected from L3 to L4 nymphs or young aphids in PBS and then fixed in 4 per cent paraformaldehyde for 30 min. Hybridization, washing and colour development were performed as described previously [15].

## 3. Results

### (a) RNA-seq analysis of the bacteriocytes

**Figure 1.**
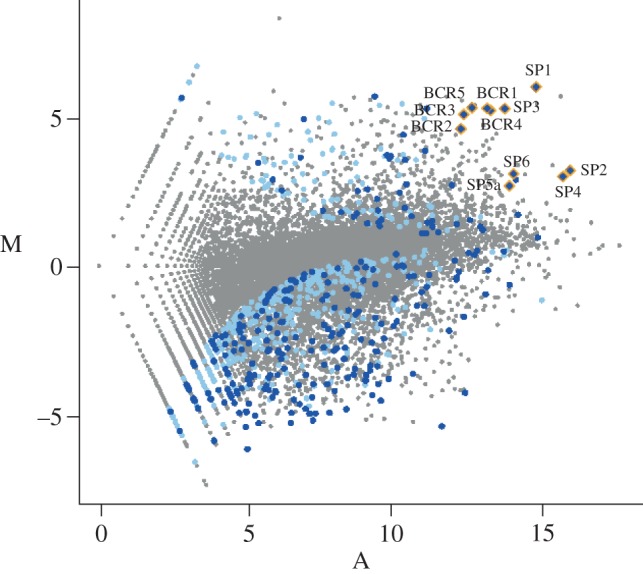
Enrichment of novel secretion proteins in bacteriocytes. Enrichment of bacteriocyte versus whole body transcripts (M) is plotted against average transcript abundance (A) on log_2_–log_2_ axes. Significantly (*z*-score > 6.0) over- and under-represented orphan genes encoding proteins with putative signal peptides (blue) and without the signals (light blue) are illustrated.

We performed deep sequencing of mRNA transcripts isolated from bacteriocytes of the pea aphid, *A. pisum*, and, for comparison, from entire aphid bodies. In total, 22 125 005 and 13 224 022 high-quality reads were generated from bacteriocytes and whole bodies, respectively. The reads were mapped to a custom set of *A. pisum* transcripts that includes pea aphid official gene set v. 1.0 (OGS1) [5] and EST sequences that, we found, were overlooked in OGS1 [11]. Out of the total number of reads for each library, 81 and 77 per cent were successfully mapped. In agreement with previous studies [5,6,8], we found that transcripts from genes involved in amino acid metabolism and those derived from lateral transfer from a non-*Buchnera* bacteria were over-represented in aphid bacteriocytes (see the electronic supplementary material, Dataset S1). We found over-expression of many orphan genes in bacteriocytes, although many orphan genes were expressed in the control sample too (see figure 1 and electronic supplementary material, figure S1A).

The N-terminus of each orphan gene product was analysed with the SignalP program to locate possible signal peptides for secretion. We found that the bacteriocyte sample contained significant enrichment for highly over-expressed transcripts that encode proteins with putative secretory signal peptides (false discovery rate < 0.01; figure 1; electronic supplementary material, figure S1C). For example, of the 30 most strongly expressed genes in bacteriocytes, 10 encode novel proteins with signal peptides.

### (b) Aphid-specific putative secreted proteins

We performed a more detailed annotation of the 11 most highly expressed orphan genes with signal sequences (those within the 50 most highly over-represented genes in bacteriocytes assessed by *z*-score) (table 1). All 11 transcripts were represented in the sequenced cDNA in our *A. pisum* 50 k full-length cDNA collection [11]. Two transcripts (BCR4 and BCR5) were missing from the official gene set of aphidbase, possibly because they encode relatively short open reading frames (207 and 231 bp respectively). While a BLASTP search of the deduced protein sequences against the arthropod protein database (see §2) and the NCBI's non-redundant protein database returned no significant hits, TBLASTN search against the EST datasets of other aphid species (*Myzus persicae*, *Aphis gosipii* and *Toxoptera citricida*) identified putative homologues for more than half of the genes examined (table 1). This result suggests that the putative secreted proteins are limited to the aphid lineage. Besides the secretion signals, no other Pfam domains were found in these proteins.

**Table 1.** Classification of novel putative secreted proteins expressed in the pea aphid bacteriocytes.

class	name	AphidBase ID	total length (amino acid)	signal peptide length (amino acid)	bacteriocyte (RPKM)	whole body (RPKM)	fold change	*z*-score rank	no. cysteine	expression pattern^a^	other aphid species^b^
*BCR family*											
	BCR4	none	69	19	2361.1	112.6	21.0	8	6	B	
	BCR1	ACYPI32128	79	23	12384.0	562.2	22.0	9	6	B	
	BCR5	none	77	19	1509.9	67.8	22.3	13	7	B	
	BCR3	ACYPI44142	86	23	6044.6	315.4	19.2	21	6	B	Mg, Ag, Tc
	BCR2	ACYPI38738	77	19	6131.9	448.3	13.7	30	6	B	
	BCR6	ACYPI49532	108	24	746.6	31.7	23.5	128	8	B	
	BCR8	ACYPI45157	67	23	811.4	117.6	6.9	238	6	B	Mp, Ag
*non-BCR*											
	SP1	ACYPI008389	108	24	12768.5	353.0	36.2	2	0	B	Mp, Ag
	SP2	ACYPI000294	178	20	10293.4	1970.1	5.2	4	0	U	Mp, Ag, Tc
	SP3	ACYPI005168	302	24	3284.8	150.3	21.9	6	0	B	Mp, Tc
	SP4	ACYPI009984	431	20	4363.0	959.2	4.5	7	0	B	Mp, Ag, Tc
	SP6	ACYPI001839	415	18	1496.4	310.0	4.8	23	0	S	Mp, Ag
	SP5a	ACYPI004796	160	19	1671.1	455.0	3.7	39	0	S	Mp, Ag, Tc

^a^B, specific to bacteriocytes in embryos and adults; S, sheath cells surrounding bacteriocytes; U, ubiquitous.

^b^EST contigs of other aphid species. Mg, *Myzus persicae*; Ag, *Aphis gosipii*; Tc, *Toxoptera citricida.* Blank cells indicate no hits in database search, but this does not necessarily mean that homologues are absent in these aphid species, because the EST datasets available for these species were small.

**Figure 2.**
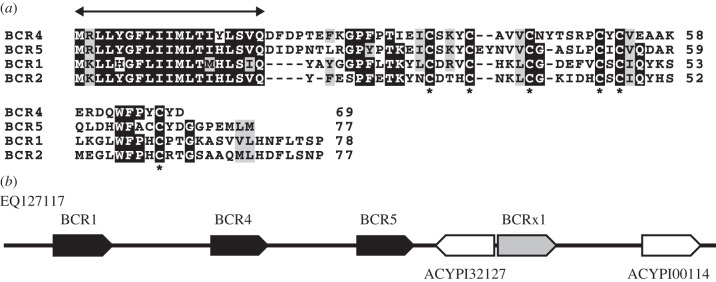
BCR1 family. (*a*) Multiple alignment of BCR1 family peptides. Identical residues are shaded black, while similar residues are shaded grey. The double headed arrow is placed above the putative signal peptide. The asterisks are placed below the cysteines in conserved positions. (*b*) Genomic organization of BCR1, BCR4 and BCR5 on contig EQ127117 are shown. The location and orientation of genes are indicated by arrows. Black and white arrows represent the BCR genes and other genes, respectively. We found a BCR-like gene, BCRx1, in the vicinity of BCR5 as represented by a grey arrow, but there is no EST evidence for this gene thus far.

The novel putative secreted proteins can be divided into two classes (table 1). One class includes genes that encode small proteins (67–108 amino acids) with six or eight cysteines and we named these bacteriocyte-specific cysteine-rich proteins (BCR family; electronic supplementary material, figure S2A). Four BCR genes (BCR1, BCR2, BCR4 and BCR5) show some similarity with each other and sequence alignment revealed a well-conserved hydrophobic amino-terminal domain, which is predicted to be a signal peptide, followed by cysteine-rich polypeptides. The cysteine-rich region is highly diverged, but the six cysteines have almost identical spacing in the predicted proteins of the four BCR genes (figure 2*a*). Three of these genes are located in a cluster within 20 kb of each other, suggesting that they may have arisen by tandem gene duplication (figure 2*b*). No similarity could be found between any of the other BCR family genes (see the electronic supplementary material, figure S2A). Comparison of BCR3 of the pea aphid with the putative homologue from the green peach aphid, *M. persicae*, showed a perfect conservation of the six cysteine residues and of their spacing (see the electronic supplementary material, figure S2B). The second family of novel proteins, which we called secreted proteins (SP family), contain no cysteines and vary in length from 108 to 413 amino acids (table 1). Over-expression of SP1 (LOC100167607) and SP3 (LOC100164129) in bacteriocytes was reported in the RNA-seq data of Hansen *et al*. [8], while other SP and BCR family genes have not been investigated in the previous studies probably because they were not annotated as protein-coding genes in the public database.

### (c) Spatio-temporal expression patterns of the novel secreted genes

**Figure 3.**
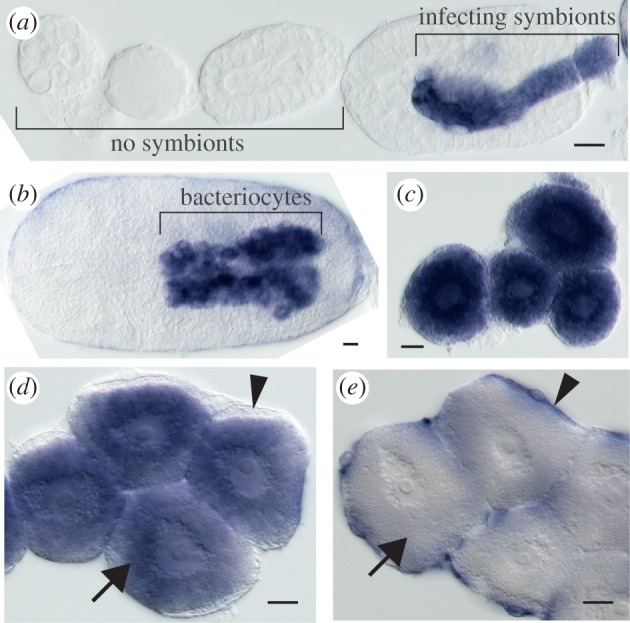
Spatio-temporal gene expression pattern of bacteriocyte-specific secreted protein. (*a–c*) Expression of BCR4, a bacteriocyte-specific cysteine-rich protein from early embryogenesis to stage 7 (*a*, left to right), in embryonic bacteriocytes (*b*), and in dissected adult bacteriocytes (*c*). (*d*) Expression of SP3 in adult bacteriocytes. (*e*) Expression of SP5a in presumptive bacteriome sheath cells. Arrows: bacteriocyte. Arrowheads: presumptive bacteriome sheath cells. Scale bar, 20 µm.

We examined the spatio-temporal expression patterns for some of the BCR and SP genes. The seven most highly expressed BCR family genes were expressed in indistinguishable patterns (see figure 3 and electronic supplementary material, figure S3). BCR family transcripts were detected first in stage-7 embryos (figure 3*a*), coincident with the incorporation of *Buchnera* into blastoderm-stage embryos [3]. At stage 7, strong expression was observed only in the syncytium that develops into bacteriocytes. Expression of all seven genes was maintained strictly in bacteriocyte cells in all later embryonic stages and in adults (figure 3*b–d*). Three of the six tested SP-family genes were expressed in patterns indistinguishable from the patterns observed for the BCR family genes (see figure 3*e* and electronic supplementary material, figure S3). These expression patterns suggest that a wide array of recently evolved secretion proteins act within bacteriocytes, perhaps to mediate the symbiosis with *Buchnera*.

Bacteriocytes are organized into organs called bacteriomes and surrounded by sheath cells [16]. Two of the six SP family genes, SP6 and SP5a, were expressed strictly in sheath cells in adults (figure 3*f*). While SP5a was expressed in fat body as well as in embryonic stages, SP6 is expressed specifically in presumptive sheath cells in the embryonic bacteriome. These sheath cell expression patterns of SP family genes suggest that novel secretion proteins may also act to help organize the bacteriome (see the electronic supplementary material, figure S4).

## 4. Discussion

In this study, we report the identification and characterization of novel families of putative secreted proteins restricted to aphid species. Lineage-specific orphan genes, which have no detectable homologues in other organisms, make up 10–20% of the genes in every animal genome sequenced to date. Although orphan genes have not been investigated intensively, they are drawing increasing attention because lineage-specific genes can account for evolution of lineage-specific traits [17,18]. This view is supported by our findings. BCR and SP genes, which show no significant sequence similarity to genes in species outside the aphid lineage, are expressed abundantly in the cells of the aphid bacteriome, where endosymbionts live. These expression patterns suggest that these orphan genes act within the bacteriome, perhaps to mediate the symbiosis with *Buchnera*.

Previous transcriptomic studies have already led to the identification of genes preferentially expressed in bacteriocytes. However, the tissue specificity and the time course of the expression of these genes have not been examined previously. In this study, whole mount *in situ* hybridization revealed that 7 BCRs and 4 SP transcripts are expressed in bacteriocytes throughout the life of the aphid. In addition, we found two transcripts encoding putative secreted protein products whose expression is restricted to sheath cells. Sheath cells form a thin casing around bacteriocytes [16,19] and, in aphids, secondary symbionts sometimes reside in sheath cells [20]. Sheath cells are observed in aphids, psyllids and many other insects [19], but little is known about the function or evolutionary origins of sheath cells. In future studies of bacteriome development and evolution, the BCR and SP genes will likely serve as valuable specific molecular markers of bacteriocytes and sheath cells.

The presence of N-terminal signal sequences in BCRs and SPs indicates that these novel proteins enter the secretory pathway. The proteins synthesized in the endoplasmic reticulum may be transported, via Golgi, to the extracellular space. Another possibility is that these proteins are targeted to *Buchnera* within the bacteriocytes. This is a probable scenario given that *Buchnera* cells are housed within vesicles constructed from host-derived membranes called symbiosomal membranes, and electron microscopic observations have shown that symbiosomal membranes are sometimes closely associated with the bacteriocyte endoplasmic reticulum [16,21]. We hypothesize that these ‘secreted’ proteins may be released into these vesicles. Recent studies in weevils and plants provide an interesting comparison with our findings in aphids. A weevil coleoptericin-A (ColA) antimicrobial peptide (AMP), which has 23-amino acid signal peptides, selectively targets endosymbionts within bacteriocytes [22]. In the *Rhizobium*-legume symbiosis, nodule-specific secreted peptides called NCRs are targeted to the bacteria [23]. In both cases, the secreted peptides regulate the proliferation of the symbionts. By analogy with these symbiotic systems, it is possible that some of the BCRs and SPs of aphids may mediate the symbiosis by direct association with the symbiont to regulate cell division and metabolic activity. The most probable target of the novel secreted peptides is *Buchnera*, but we cannot exclude the possibility that they interact with secondary symbionts and microbial intruders.

The BCR family genes may have evolved from *defensin*-type AMPs, small proteins that contain six to eight cysteines, which are common in both animals and plants. AMP-like secretory cysteine-rich proteins have evolved in various taxa and are involved in diverse cell–cell communication functions, including symbiosis [24]. Notably, the evolution of BCR genes in aphids mirrors the evolution of cysteine-rich secreted proteins in leguminous plants. The *Medicago truncula* genome contains a family of novel genes encoding AMP-like cysteine-rich proteins with secretion signals [25,26]. More than 300 genes of this family are expressed specifically in root nodules, the symbiotic plant organs that house nitrogen-fixing endosymbiotic bacteria, and at least some of these proteins target bacteria and induce differentiation [23]. The remarkable convergence of protein sequence composition and expression pattern for these genes between leguminous plants and aphids suggests that there may be common principles underlying evolution of endosymbiosis in divergent taxa.
